# Complete genome sequence of *Enterococcus faecalis* phage EF_RCK

**DOI:** 10.1128/mra.00109-24

**Published:** 2024-04-23

**Authors:** Loh Michelle JiaMin, Priyadarshini Karthikeyan, Ramesh Kumaresan, Andrew Millard, Sivachandran Parimannan, Heera Rajandas

**Affiliations:** 1 Centre of Excellence for Omics-Driven Computational Biodiscovery, AIMST University, Kedah, Malaysia; 2 Faculty of Dentistry, AIMST University, Kedah, Malaysia; 3 Department of Genetics and Genome Biology, University of Leicester, Leicester, United Kingdom; 4 Center for Evolutionary Hologenomics, Globe Institute, University of Copenhagen, Copenhagen, Denmark; Queens College, Queens, New York, USA

**Keywords:** *Enterococcus faecalis* phage, genome

## Abstract

A lytic bacteriophage EF_RCK infecting *Enterococcus faecalis* was isolated from a water sample collected in a raw cockle storage container at Taman Ria market, Sungai Petani, Malaysia. The phage has a 57,848-bp double-stranded DNA genome harboring 107 protein-encoding genes and shares 90.9% nucleotide similarity with *Enterococcus* phage EFKL (*Saphexavirus* genus).

## ANNOUNCEMENT

*Enterococcus faecalis*, an opportunistic pathogen, is progressively implicated in various oral diseases (1). The intrinsic resistance of *E. faecalis* to several commonly used antibiotics and its biofilm formation capacity have posed significant challenges in controlling infections caused by this bacterium (2). Considering phage therapy as a promising alternative to antibiotics and effective in eradicating biofilms, we present the complete genome sequence of *Enterococcus* phage EF_RCK capable of infecting against biofilm-forming *E. faecalis*.

Phage EF_RCK was isolated from a water sample obtained in a raw cockle storage container at Taman Ria market in Sungai Petani, Kedah, Malaysia (5.6667^o^N, 100.5041^o^E). Briefly, 10 mL of the raw water sample was enriched with 200 µL of the overnight *E. faecalis* ATCC 29212 culture in 10 mL brain heart infusion broth containing 50 µL of 1 M calcium chloride (CaCl_2_). The mixture was incubated at 37°C for 24 h. The enrichment was centrifuged and filter-sterilized through a 0.22-µm syringe filter before subjecting to clonal purification for five successive rounds using the soft agar overlay method (3). Phage DNA was extracted from the purified phage lysate using phenol-chloroform (4). The library was constructed using unsheared and non-size selected DNA with a native barcoding kit (SQK-NBD112.24) from Oxford Nanopore Technologies. Sequencing was performed using the SpotON flow cell (R10.4.1) on the MinION platform, and high-accuracy base calling was performed using Guppy v6.5.7. A total of 3,536 raw reads with a read length *N*_50_ of 2,633 were generated and trimmed using Porechop v0.2.4 (5). The quality of the trimmed reads was assessed using Nanoplot v1.41.6 (6). The remaining 3,479 reads were assembled *de novo* using Flye v2.9.1-b1780 (7) into a single contig and further polished with Medaka v1.8.1 (https://github.com/nanoporetech/medaka). Genome annotation was carried out using Prokka v1.12 (8) with the PHROGs database (9). PhageLeads tool was used to screen lysogenic, virulence and antibiotic resistance genes in the genome (10). Default parameters were applied for all the software tools unless otherwise specified.

The genome of phage EF_RCK consists of 57,848 bp with a GC content of 39.9% and 125.8× coverage. CheckV v1.0.1 predicted that the genome has a completeness of 100%, with a high confidence level (0%–5% error) (11). A total of 107 CDSs were identified, of which 45 were annotated as known functional genes, including 1 tRNA gene. Additionally, no lysogenic lifestyle, antimicrobial resistance, or virulence genes were detected in the genome. BLASTn (https://blast.ncbi.nlm.nih.gov/Blast.cgi) search of the complete genome against the nonredundant NCBI database (accessed 1 February 2024) revealed that phage EF_RCK shared the highest nucleotide similarity of 90.9% with *Enterococcus* phage EFKL (GenBank accession number OP831581) which belong to the genus *Saphexavirus*, as computed by the VIRIDIC web service (accessed 1 February 2024) (12). According to the International Committee on Taxonomy of Viruses standard (13), phage EF_RCK is, therefore, classified as a double-stranded DNA virus from the genus *Saphexavirus*.

## Data Availability

The complete genome of phage EF_RCK has been deposited in the GenBank database under the accession number PP028463. The associated BioProject, SRA, and BioSample accession numbers are PRJNA1070424, SRR27778910, and SAMN39645757, respectively.
